# Modified—modified radical mastoidectomy

**DOI:** 10.1007/s00405-023-08021-w

**Published:** 2023-05-17

**Authors:** Deviprasad Dosemane, Meera Niranjan Khadilkar, Navya Parvathareddy

**Affiliations:** Department of Otorhinolaryngology and Head and Neck Surgery, Kasturba Medical College, Mangalore, Manipal Academy of Higher Education, Manipal, 575001 India

**Keywords:** Mastoid, External auditory canal, Fistula, Mastoidectomy

## Abstract

**Purpose:**

It is unusual to have communication from the external auditory canal (EAC) directly to the mastoid, totally sparing the tympanum. These patients need a different surgical approach, a modified canal wall-down procedure, to completely clear the disease but fully preserve the tympanum. We present one such exceptional case.

**Case presentation:**

A 28-year-old lady presented with ear discharge for 1 year. Imaging confirmed the canal-mastoid fistula, but the entire tympanum was normal. We performed a modified-modified radical mastoidectomy.

**Conclusions:**

Canal-mastoid fistula is an infrequent entity and may be idiopathic. Despite being evident on clinical examination, imaging aids in assessing size and location of the defect. Although EAC reconstruction may be attempted, the majority require a canal wall-down procedure.

## Introduction

Chronic otitis media (COM) is a common cause of ear discharge. Squamous type of COM is more likely to result in spread of disease from middle ear to mastoid and bony erosion of the scutum, and in a few cases the entire posterior canal wall; bone destruction is not known to occur in mucosal type of COM [1]. External auditory canal (EAC) cholesteatoma is associated with localized erosion of the posteroinferior canal wall and progression to the mastoid cavity [2, 3]. Other causes for posterior canal wall defects include aggressive keratosis, radiation, osteonecrosis due to immunosuppression or malignancy, and iatrogenic or idiopathic [4, 5]. We came across an unusual case of canal-mastoid fistula with symptoms resembling those of mucosal COM, however, with an intact tympanic membrane (TM).

## Case report

**Fig. 1:**
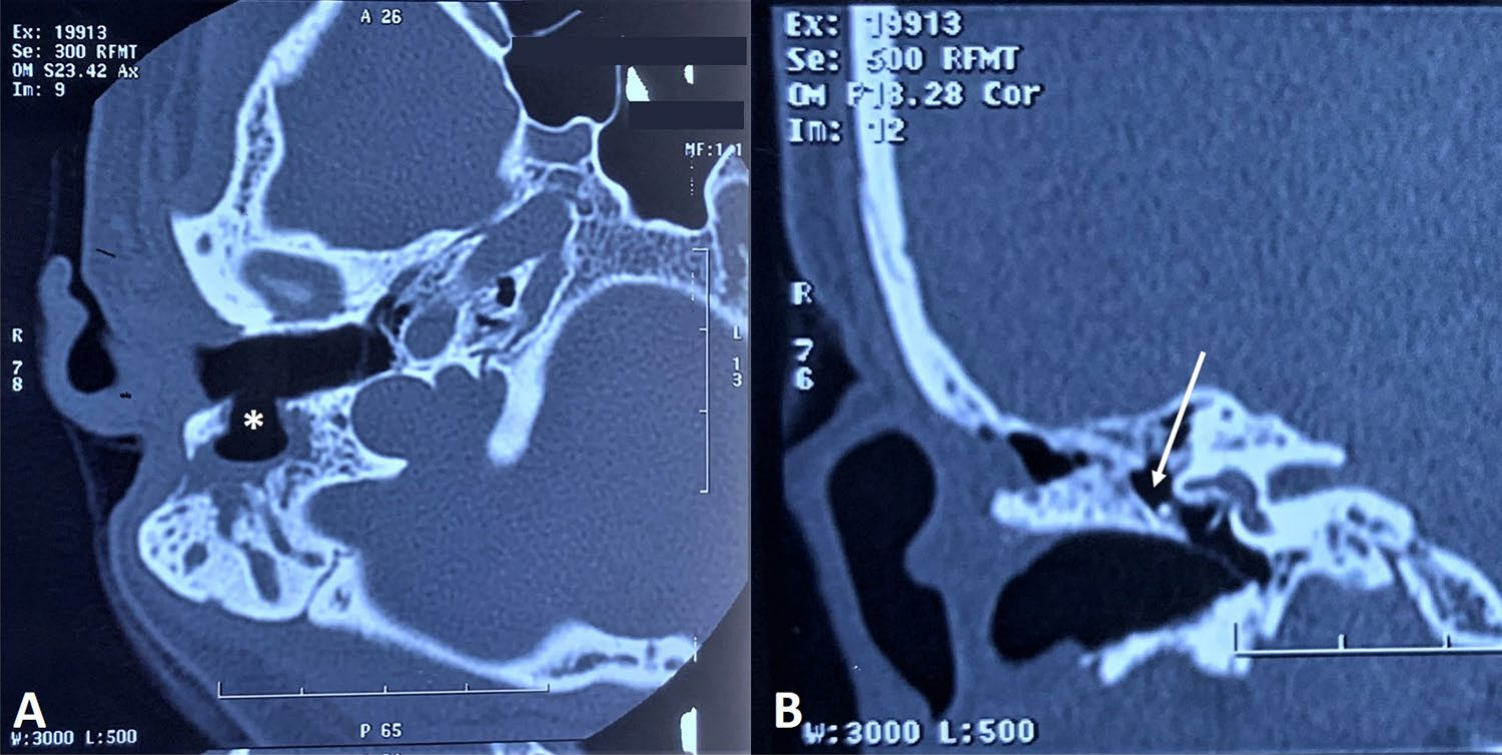
HRCT temporal bone **A** axial view showing the canal-mastoid fistula (*) and **B** coronal view showing normal mesotympanum and attic (white arrow)

**Fig. 2:**
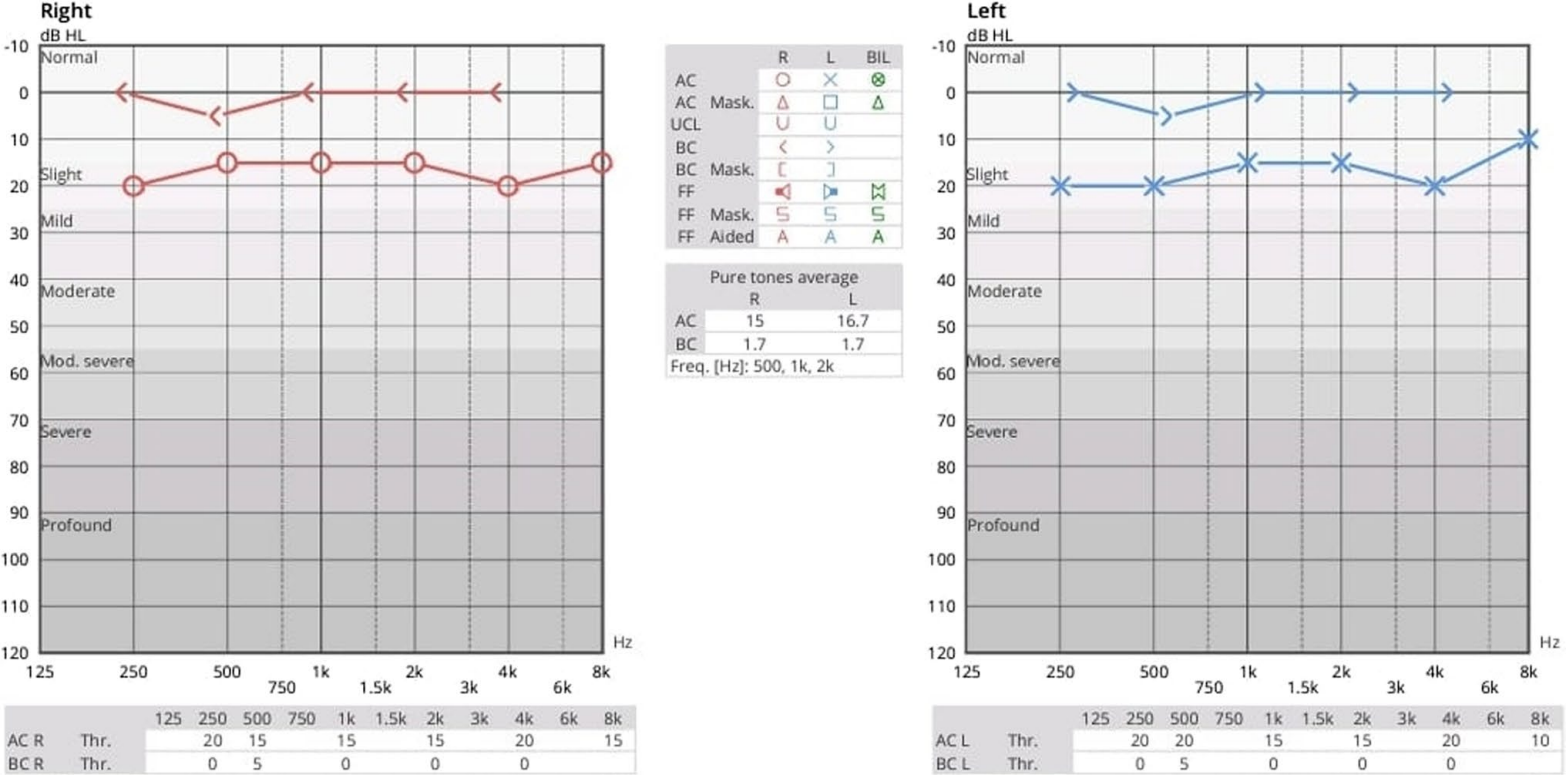
Preoperative pure tone audiometry

A 28-year-old lady presented with intermittent profuse non-foul smelling, non-blood-stained mucopurulent discharge from the right ear for one year, often associated with upper respiratory tract infection. The patient complained of reduced hearing limited to the episodes of ear discharge. She gave no history of pain or itching in the ear, trauma or surgery. She had no complaints in the left ear. Examination showed mucopus discharging from the mastoid to the EAC, with an intact TM. The canal-mastoid fistula had regular margins; no granulation tissue or cholesteatomatous flakes were noted at the fistula or the mastoid. On the left side, the posterosuperior wall of EAC was widened and thinned out, lined by normal epithelium, with no granulation or discharge. High-Resolution Computed Tomography (HRCT) temporal bone confirmed the canal-mastoid fistula, with soft tissue density in the mastoid cavity (Fig. 1); the entire TM, middle ear cavity and its contents including the ossicles were normal. Pure Tone Audiometry (PTA) showed normal hearing bilaterally (Fig. 2).

**Fig. 3:**
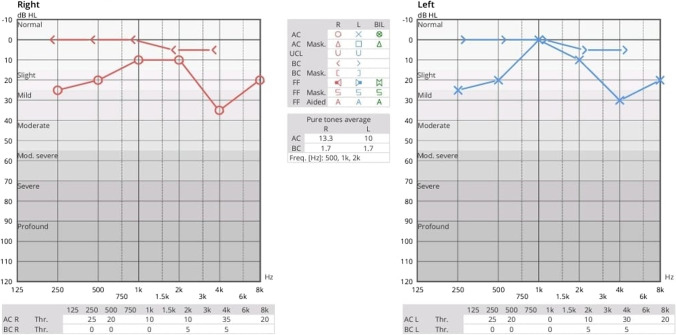
Postoperative pure tone audiometry

**Fig. 4:**
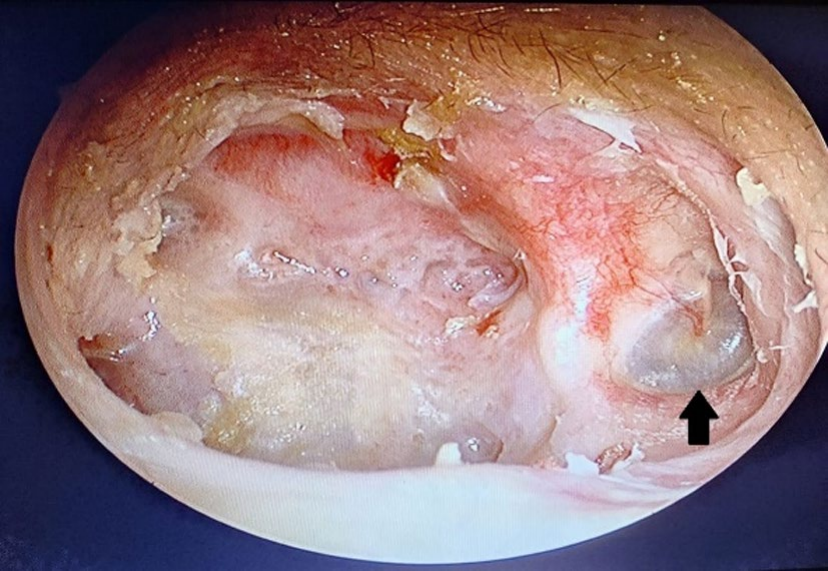
Postoperative endoscopic image of the right ear

The patient underwent right cortical mastoidectomy and lowering of posterior canal wall while retaining superior canal wall, scutum, TM, middle ear cavity and its contents. Aditus was blocked with a piece of conchal cartilage; temporalis fascia was used to cover the cartilage and the rest of the cavity. Concho-meatoplasty was performed and the wound was sutured in layers. This is a modification of modified radical mastoidectomy; hence, the name. Intraoperative and postoperative periods were uneventful. Postoperative hearing was also normal (Fig. 3). The patient was regularly followed up and the results are promising so far. (Fig. 4).

## Discussion

Approximately 70% of the patients with COM present with ear discharge [1]. The occurrence of canal-mastoid fistula with ear discharge is a rare entity. Canal-mastoid fistula is a known complication of canal wall-up mastoidectomy. Agan noted 88% of such fistulae in patients with history of middle ear surgery, of which 87% had undergone a canal wall-up mastoid procedure [6]. However, our patient gave no history of ear surgery or trauma. The TM was intact, similar to the findings of previous studies (53%) [6].

**Fig. 5:**
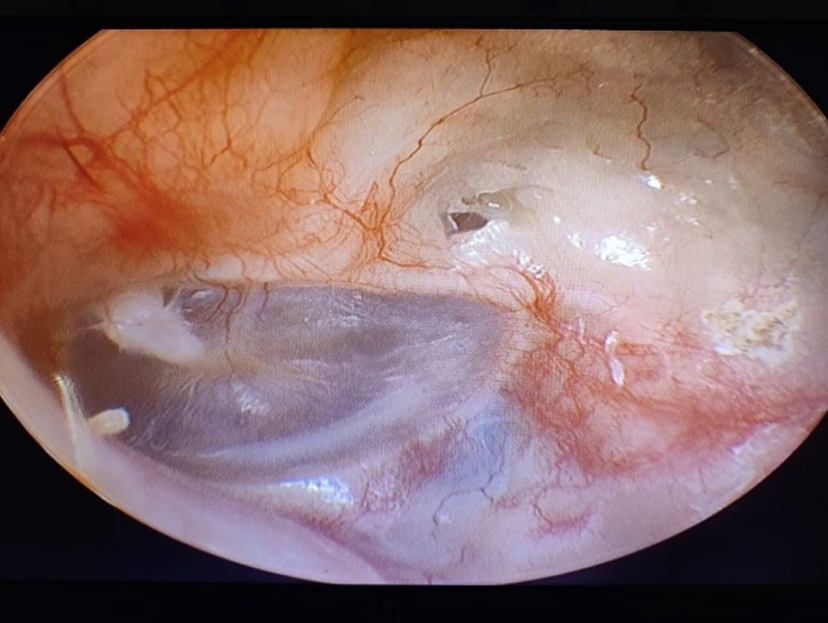
Endoscopic image of the left ear

Studies show that while keratosis obturans progresses through TM to the tympanum, EAC cholesteatoma progresses to the mastoid by destroying the posterior EAC [3, 7]. Our patient did not give history suggestive of either of these two diseases, thus ruling them out. Her left ear (Fig. 5) showed widened and thinned-out posterosuperior part of EAC with a normal epithelial lining, without granulation or discharge, possibly depicting the onset and progression on the right side.

HRCT temporal bone aids in assessing the defect size and location and ruling out active pathology in the middle ear or mastoid [4]. Agan studied 17 cases of canal-mastoid fistulas; CT was performed in 82% of cases, all of which demonstrated the fistula [6]. HRCT demonstrated the fistula in our patient too.

These defects necessitate closure to achieve respite from ear discharge and to avert retraction pockets and cholesteatoma formation [4]. Materials used for closure include alloplastic ones such as titanium, hydroxyapatite and silastic. They are easily available in various shapes and sizes and without donor morbidity. However, vascularization may be hindered, and spontaneous extrusion may occur due to biofilm formation. Bony sigmoid sinus plate may be used for larger defects, but caution must be taken to avoid venous bleeding during lifting the bony plate over the sinus [8]. Tragal cartilage with intact perichondrium may also be used [4]. Canal wall-down mastoid surgery with or without EAC reconstruction is another treatment option [6, 7].

Bondy first described modified radical mastoidectomy in 1910, wherein the superior bony meatal wall and a part of the posterior bony meatal wall were removed without disturbing the TM, ossicles or middle ear cavity. However, persistence or recurrence of ear discharge due to incomplete clearance of infected mastoid air cells, and squamous debris accumulation due to incomplete clearance of tip cells and high facial ridge led to Bondy’s technique losing favour as the preferred technique. Jansen, in 1958, described intact canal wall tympanomastoidectomy that involved mastoid air cell exenteration and opening of the facial recess to gain access to the middle ear. This technique also allowed TM reconstruction, thus improving postoperative hearing [9]. In my experience of close to two decades in the field of otology, this was the third case with canal-mastoid fistula. The first case with intact TM underwent cortical mastoidectomy with posterior bony canal wall reconstruction with periosteum lined, lateral bone plate of mastoid cortex, carefully preserved and drilled out during the initial stage of cortical mastoidectomy. This bone with periosteum graft was augmented by temporalis fascia. However, this failed after a year, and he had to undergo a canal wall-down procedure. The second case had a canal-mastoid fistula with TM perforation. She underwent tympanoplasty, cortical mastoidectomy and reconstruction of the posterior EAC with conchal cartilage plus perichondrium on both sides. However, this too failed within a year, and the patient underwent a canal wall-down procedure. Keeping in mind my previous encounters, I opted not to preserve the posterior EAC and the outcome was successful.

## Conclusion

Canal-mastoid fistula is an infrequent entity and may be idiopathic. Despite being evident on clinical examination, HRCT aids in assessing size and location of the defect. Although EAC reconstruction may be attempted, the majority require a canal wall-down procedure.

## Data Availability

Data transparent.
